# An advanced and efficient asymmetric PCR method for microarray applications

**DOI:** 10.3389/fbioe.2022.1045154

**Published:** 2022-11-30

**Authors:** Suresh Reddy Banda, Holger Klapproth, Nicolaas Smit, Sonja Bednar, Thomas Brandstetter, Jürgen Rühe

**Affiliations:** ^1^ Laboratory for Chemistry and Physics of Interfaces, Department of Microsystems Engineering (IMTEK), University of Freiburg, Freiburg, Germany; ^2^ Safeguard Biosystems Holding Ltd., London, United Kingdom

**Keywords:** asymmetric polymerase chain reaction, single stranded DNA, amplification, microarray, hybridization

## Abstract

The sensitivity of a PCR based biochip assay relies on the efficiency of PCR amplicons in binding to the microarray spots. The essential factor determining the sensitivity is the amount of single stranded (ss) amplicons available for biochip hybridization. Asymmetric PCR can generate ss-amplicons depending on the ratio of primers used in the amplification process, but this process is often inefficient. We report a novel variant of PCR called the Asymmetric Exponential and Linear Amplification (AELA) which can overcome these issues and generate large amounts of single stranded amplicons. AELA-PCR introduces an amplification strategy that makes use of both exponential and linear amplification of the target nucleic acid. This is done by specifically designed primers and choice of adequate thermal profiles. In conventional PCR with a classical thermal profile, these specifically designed primers will work normally and contribute to an exponential increase of amplicons. A designed sequence extension of one of the primers and a very specific thermal profile, will result in a situation that the extended primer will be the only functional one for amplification, resulting in a linear phase of the amplification process. That is why during this step only one of the two strands of the target is amplified linearly and no longer exponentially. The result of the whole process is an amplification product enriched very strongly in one of the two single strands of the target. These adaptions in PCR are particularly favorable where the generation of ss-DNA/RNA is required. We demonstrate the higher biochip sensitivity of AELA-PCR compared to conventional amplification methods with an example of the *Staphylococcus aureus* detection on a DNA oligonucleotide microarray.

## Introduction

Microarrays are one of the applications that have received significant attention in conventional PCR end-point detection based methodologies. Their fast and highly parallel analysis procedures encouraged a broad use of this technology in medical diagnostics (Miller and Tang, 2009). The key in success of a microarray system lies in the efficient hybridization of the PCR products or amplicons onto the surface attached capture probes (Koltai and Weingarten-Baror, 2008). A PCR system that employs equal concentrations of the primers used for amplification is called a “symmetric PCR.” This results in exponential amplification of the target DNA and generation of double stranded (ds) amplicons. It is well known that hybridization with ds-amplicons results in lower hybridization efficiencies to microarrays. This is due to reannealing of amplicons leaving low amounts of ss DNA available for hybridization to probes (Rizzi et al., 2017). Even after prolonged incubation time only a fraction of the total number of amplicons generated, can bind to the array, resulting in significantly lowered assay efficiencies (Deregt et al., 2006). On the other hand varying the temperature to increase the number of ssDNA molecules rarely poses a feasible option as this also influences the binding of amplicons to the surface attached probes, especially in multiplexed assays (Zhu et al., 2007).

An improved PCR technique for the generation of single stranded DNA called “asymmetric PCR” has been described by Gyllensten and Erlich. (1988). It uses unequal concentrations of the primers for amplification, typically in the range of 1:5 to 1:100. During the early stages of amplification, as long as both primers remain available, the amplification occurs exponentially as the symmetric PCR generates ds-amplicons. Once the lower concentrated, i.e. the limiting, primer is depleted in the reaction mix, only the still available excess primer is able to continue the amplification of target DNA and thereby produces single stranded amplicons. It has been shown that this allows improved sensitivities in microarray-based analytics because of higher diffusion rates and binding efficiency of single stranded amplicons to the capture probes (Gao et al., 2003). However, moving to a more or less exclusively linear process renders the amplification extremely slow. Additionally, the primer design criteria used for asymmetric PCR are typically similar to those of a conventional symmetric PCR. However, when the reaction is controlled by having only a low concentration of one of the primers, all events impacting primer availability like primer dimer formation or even low levels of unspecific binding can lead to large variations of the primer content at the linear phase. This results in problems with the reproducibility of amplification process. In addition, the transition between the exponential and the linear phase when the concentration (c) of the limiting primer is approaching c = 0 is not well defined. This leads to problems in predicting the number of ss-amplicons formed (Poddar, 2000).

In this paper we describe an improved asymmetric PCR method, called Asymmetric Exponential and Linear Amplification PCR (AELA-PCR) which generates in a very simple way large amounts of ss-amplicons. It employs a straight-forward amplification strategy and specifically designed primers that generate ss-amplicons with predictable kinetics. We demonstrate the principles of this new asymmetric PCR method by amplification and detection of known concentrations of *Staphylococcus aureus* genomic DNA spiked with human DNA. This is then validated with spiked blood samples with known concentrations of bacterial load and clinical samples.

## Materials and methods

### Genomic DNA

The target DNA is obtained from different sources according to the study design. For the initial proof-of-principle and guard banding studies of AELA PCR, pure genomic DNA of *Staphylococcus aureus* (DSM 20231) bacterial strain is obtained from Deutsche Sammlung von Mikroorganismen und Zellkulturen (DSMZ, Germany). Copy number dilutions are prepared with this DNA by calculating molecular weight from the genome size of the respective strain as calculated in Table 1.

**TABLE 1:** Calculation of dilution factor for preparing genomic copy number dilutions from genome size and molecular weight of respective species a detailed table is provided as Supplementary Table S3.

Species	Genome size (bp)	Mol wt (g/mol) [genome size X 660]	Mol wt in pg/1 copy	Mol wt in pg/10,000 copies	Stock (pg/µl)	Vol. req. To make 10,000 copies from stock	Water to add (final 50 μl)
*Staphylococcus aureus DSM 20231*	2782560	1836489600	0.0031	30.5065	200	7.63	42.37

For validation and Limit of Detection (LoD) studies, the extracted genomic DNA from bacterial culture spiked blood (commercially sourced as human whole blood K2-EDTA from Lampire Biological Laboratories, United States) is used. The spiked blood samples are prepared by adding a known concentration of *Staphylococcus aureus* ATCC 12600 bacterial culture into the blood which is then run through bead beating followed by extraction system. The human genomic DNA which is an extract from human whole blood (as mentioned above) is used in PCR reactions involving pure genomic DNA, as a background material. This way of sample processing mimics that of clinical samples to some extent and aims to test the developed amplification system. The extracted human DNA and *Staphylococcus aureus* genomic DNA from spiked blood samples is prepared as described by Long et al., 2014 and Klapproth and Smit al, 2018 and provided by Safeguard Biosystems Holdings Ltd.

### Primer design

**FIGURE 1:**
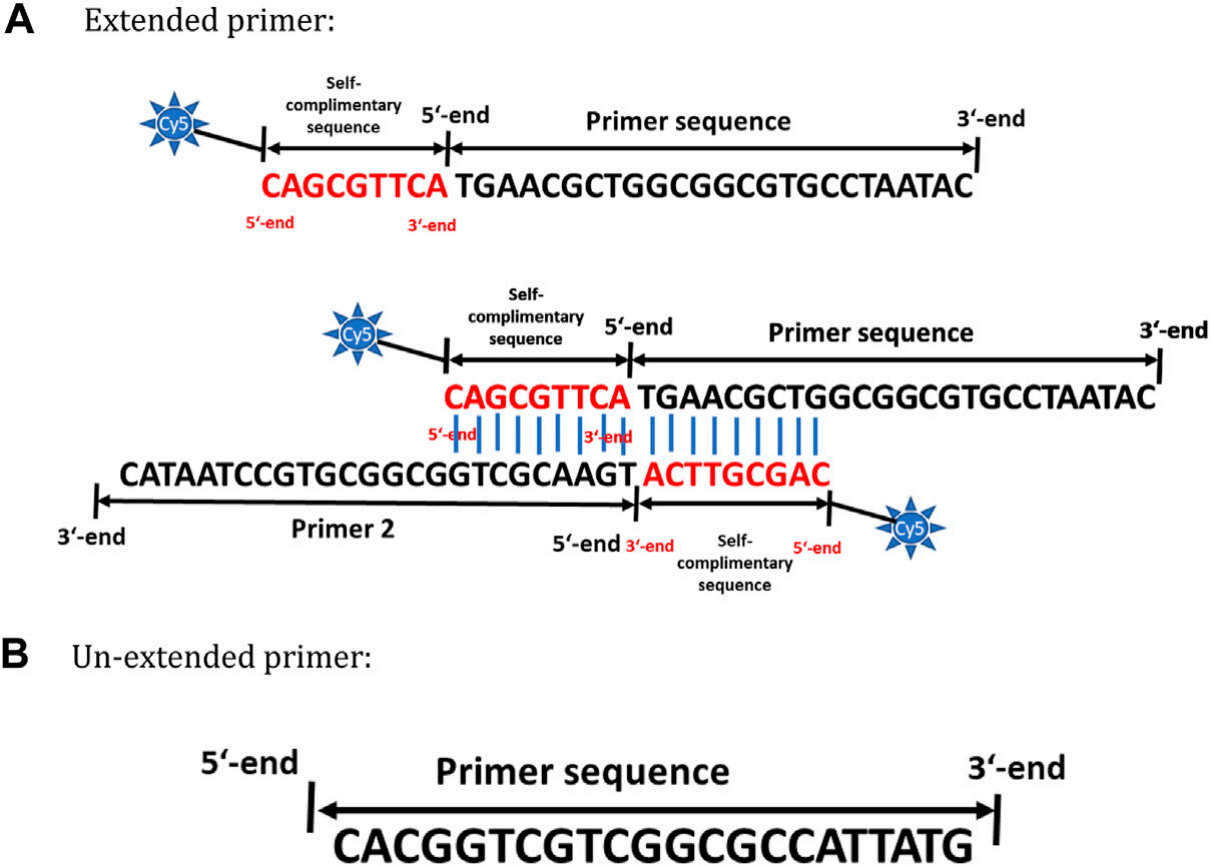
Primer design strategy for AELA PCR. **(A)** Schematic representation of the inverted self-complementary tail sequence at the 5′ end of extended primer sequence. The first ninebases represented in red are complementary to the first nine bases in order to allow self-hybridization of the primer. The blue lines represent the hydrogen bonding between the nucleotides. **(B)** Schematic representation of the un-extended primer sequence.

The primer pair for amplifying *Staphylococcus aureus* is designed to target a 498-bp length covering variable regions V1, V2, and V3 of its 16S rRNA gene. A recent study by Walker et al., 2020 is in line with our studies (not shown in this manuscript) in understanding the importance of using bacterial 16S variable regions for differentiation and specific identification. In short, the primer sets designed in these regions show very little or almost no off-target amplification. A detailed description of primer design strategy is explained in “Principle of AELA PCR” sub section in chapter results and discussion. Figure 1 represents the primers designed and tested for amplifying 498-bp 16S rRNA region of *Staphylococcus aureus*. The Tm values are calculated according to the nearest neighbour model from OligoCalc by Kibbe, 2007. Same primers were used excluding the self-complimentary region for conventional PCR (symmetric PCR) and asymmetric PCR methods during their comparison studies with AELA-PCR.

### PCR conditions

A commercial i-Taq 2X PCR master mix kit from Intron Biotechnologies Inc., (South Korea) was used for amplification. The kit contains 2.5 IU/μl of DNA polymerase, 2.5 mM each of dNTP’s and 1X reaction buffer with 3 mM MgCl_2_. For experiments with target DNA from pure *Staphylococcus aureus* cultures, a standard 20 μl reaction is made with 10 μl of i-Taq mix, 1 μl of primer mix, 2 μl of extracted human genomic DNA, 6 μl of nuclease free water and 1 μl of template DNA is used. For reactions with clinical blood samples, 4 μl of sample volume was used and water is adjusted accordingly. For no template controls, the target template was replaced by water. The concentrations of primers were adjusted according to the type of PCR reaction. For a symmetric PCR reaction, 2 μM each of the extended and un-extended primer is added. Whereas for an asymmetric and AELA PCR reactions, the extended and un-extended primers were added at a ratio of 20:1. The amplification reactions were performed using the Veriti 96-well thermal cycler from Applied Biosystems. In order to achieve amplification and labelling of the amplicon in a single step, the extended primer is labelled with Cy5 at its 5′-end. This enables the amplicons to be ready for use in microarray applications. The following thermal profile is used for symmetric and asymmetric PCRs: 95°C for 2 min, 50 cycles of amplification with 95°C for 20 s, 58°C for 15 s, 72°C for 40 s followed by a final extension at 72°C for 2 min. A similar thermal profile is used for AELA PCR with an additional amplification stage with 95°C for 20 s and 72°C for 50 s. However, the number of amplification cycles for each stage of AELA PCR is decided according to the guard banding studies.

For experiments with SYBR green to calculate the efficiency of primers, commercially available ABS PowerupTM SYBR Green 2x Master Mix kit from Applied Biosystems containing 3.5 IU/μl of dual hot-start Taq DNA polymerase, 5 mM of each dNTP’s, 1X reaction buffer with ROX as passive reference dye and 5.5 mM of MgCl_2_ was used. For a typical 20 μl reaction, 10 μl of SYBR Green Taq mix, 1ul of primer mix (with 0.5 μM each of forward and reverse primers), 2 μl of template DNA and 7 μl of water were used. For blank controls water was added instead of target template. The amplification reactions were performed on an Applied Biosystems-9500 thermocycler using a 0.2 ml MicroAmp Optical 96-well reaction plates (Applied Biosystems). The following PCR program was used: initial denaturation at 95°C for 2 min, 40 cycles of amplification and data collection phase with 95°C for 20 s, 58°C for 15 s, 72°C for 40 s followed by a final extension at 72°C for 2 min. Melt curve analysis of the amplicons to investigate possibility of unspecific amplicons was done with 95°C for 15 s with readings at 0.3°C temperature interval from 40°C for 1 min to 95°C for 15 s followed by final extension at 60°C for 15 s.

### Microarray fabrication

Microarrays were fabricated according to the procedure described by Rendl et al., 2011 using the single one-step immobilization technique with a photo-reactive copolymer containing (N, N-dimethylacrylamide–DMAA at 92.5 mol%, methacryloyloxy benzo-phenone–MaBP at 5 mol%, and Na-4 styrenesulfonate–SSNa at 2.5 mol%). A typical array spot mix was prepared with 350 mM sodium phosphate buffer—NaPi (pH8), 20 μM probe oligonucleotide, 1 μM of Cy3 oligo and 1 mg/ml of photoreactive copolymer. The print solution is then spotted into each well of unmodified polystyrene 96 well microtiter plates from Greiner Bio One, Germany, using a contactless print procedure with SciFlexArrayer S5—Scienion AG, Germany. The use of 96 well plates allows to increase the throughput of the assay. Spots were printed with a PDC90 nozzle as three drops per spot resulting in 1.2 nl (approx.) of volume per spot at 22°C temperature and 65% relative humidity. Once the printing is done, the microarray plate is then oven dried at 65°C for 1 h and irradiated with 1J/cm^2^ of UV light at 254 nm using the UV Stratalinker 2,400 (Stratagene, United States). This enables the crosslinking of the co-polymer, which in turn immobilises the microarray spot on to the polystyrene surface (one-step photo-crosslinking and immobilization technique). The unbound probes and polymer is washed with 1 mM NaPi (pH-8) buffer using a 96-well microplate washer (Anthos Fluido from Biochrom UK). For hybridization, the amplicons from PCR were incubated with 125 mM NaPi (pH-8) in a two-step process that includes denaturation at 80°C and 350 rpm for 10 mins and hybridization at 55°C and 350 rpm for 30mins on a Bioshake heater shaker (Q Instruments GmbH, Germany). After hybridization, the plates are washed with 1 mM NaPi buffer (pH-8) to remove excess and unbound PCR product and scanned for fluorescence intensities using a SensoSpot Microarray analyser (Sensovation AG, Germany).

## Results and discussion

### Principle of AELA PCR

The key concept of the AELA-PCR is its embodiment of two steps of amplification, the initial exponential and the following linear step. These exponential and linear amplifications are achieved by an appropriate primer design that allows the use of different amplification temperatures at each stage. The initial exponential amplification is performed at its optimal annealing temperature followed by the linear amplification which is performed at a more elevated temperature. To this the primers used for amplification are designed to have different melting temperatures (T_m_). One of the primers of the system is designed to have a longer nucleotide sequence by adding an inverted complementary sequence at the 5′ end with first nine nucleotides of the primer sequence, as shown in red in Figure 1 to achieve higher T_m_. This primer with higher T_m_ is delineated as the “extended primer” (Figure 1A). The preferable length of the extended primer is between 25 and 30 bases excluding the self-complimentary tail whereas the overall T_m_ of the primer should be around 68–70°C. The second primer is designed according to standard primer design rules with a length of 15–30 bases and a lower T_m_ of around 55–50°C compared to the extended primer. This is delineated as the “un-extended primer” (Figure 1B). The overall T_m_ of the extended primer should preferably be at least 6°C higher than the un-extended primer.

The inverted self-complimentary tail sequence on the extended primer is designed with three features: the inverted repeat forms primer dimers at low temperatures stopping them from binding to templates or secondary templates with low homology which would generate unspecific amplicons. Secondly, the overhanging 3′-end of a dsDNA or a primer dimer cannot act as a template for *Taq* DNA polymerase (Brownstein et al., 1996) which also prevents unspecific amplification at lower temperatures so that the function is similar to a “Hot-Start” primer. Finally, the overall primer length is long which increases the T_m_ of the primer. As this tail sequence is also not a special external sequence, from a principal point of view it can be adapted to any primer of interest that is intended to be used for AELA PCR. Nevertheless, the number of bases to be used for inverted self-complimentary tail sequence has to be carefully determined according to the T_m_ requirements.

To further simplify the process for microarray applications, a single one-step labelling and amplification strategy is followed in the AELA-PCR system. This is achieved by labelling the ‘extended primer’ with cyanine dye at its 5′-end which eventually is incorporated to the amplicons during the amplification procedure. This excludes the laborious and time consuming post-amplification labelling process used in routine microarray procedures to generate labelled PCR amplicons (Ouellet et al., 2009; Taitt et al., 2019). The efficiency of such designed primers reported in this study are tested by 5-fold *Staphylococcus aureus* template dilution amplification curve and melt curve analysis of amplicons. The data suggested an amplification efficiency of 99.6% (refer to Supplementary Figures S2–S4; Supplementary Table S2).

**FIGURE 2:**
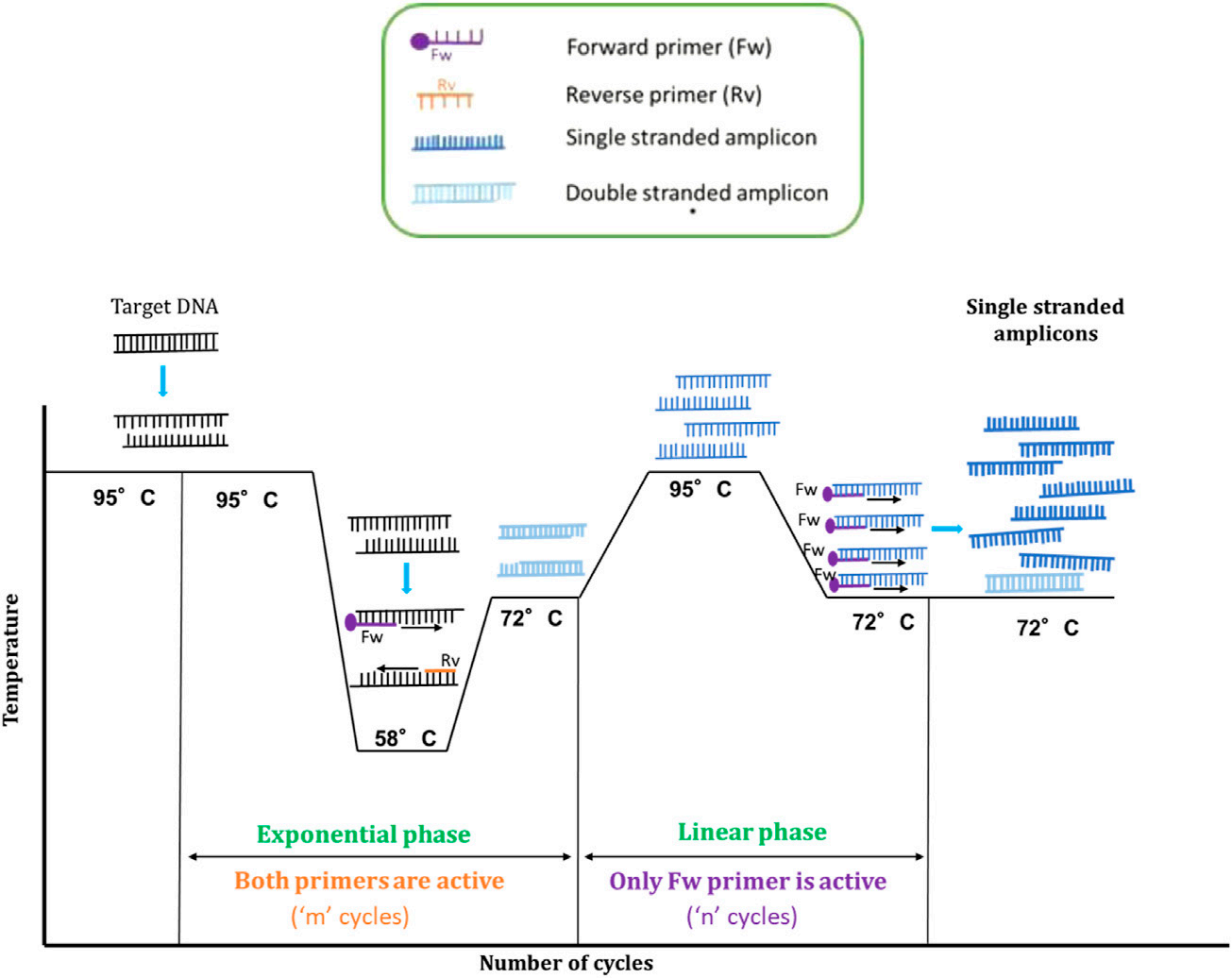
Schematic representation of AELA-PCR thermal profile. The target ds-DNA is denatured and then annealed at 58°C to bind with Forward (Fw) and Reverse (Rv) primers in order to synthesize new complementary strands. These new strands are extended resulting in two ds-DNA fragments. The new strands are themselves extended at the side where the extended primers had been elongated to get an inverted self complementary tail sequence. The newly obtained strands are then denatured and reannealed for the linear phase at a higher temperature than the annealing temperature of the exponential phase. As a consequence, only the extended primer will be active for sequence elongation during the linear phase. It means that as a whole, the resulting product will be enriched in single stranded DNA of one the strands of the DNA target (single stranded amplicons are depicted in dark blue and double stranded amplicons in light blue). The required amplification cycles at each phase “m” and “n” can be balanced as discussed in guard banding studies.

Using the above mentioned primer design criteria, the cycling patterns for amplification is then organised with an intention to generate more single stranded amplicons by introducing a linear phase of amplification after a conventional exponential amplification. After the initial denaturation 95°C, the template is annealed with the primers at 58°C. At this temperature, both the extended and un-extended primers are expected to be functional and bind to the target DNA enabling amplification of sense and anti-sense strands (Figure 2). After the primer annealing, the initial extension phase is kept short only for 30 s. This is similar to that of a basic PCR reaction in generating amplicons from both the strands at an exponential rate.

The double stranded amplicons from the exponential amplification are then denatured again at 95°C and reannealed at higher temperature (72°C) than in the initial exponential phase (58°C). The respective temperature being in the optimal activity range of the Taq polymerase (Lawyer et al., 1993; Terpe, 2013)—besides facilitating the annealing of primers, the elongation of the strands is done simultaneously. To perform all these activities in one stage, a longer duration of 50 s is allocated for this stage. The second annealing temperature has to be chosen such that it is at least 6°C higher than the T_m_ of un-extended primer. With these conditions only the extended primer, which has a higher T_m_ stays active and continues amplification by binding to sense or anti-sense strand of the target DNA. The amplification proceeds at a linear rate by using only one primer and encourages the generation of ss-amplicons. Even if the un-extended primer is still present, at this temperature it will not amplify the DNA as it cannot bind to the target DNA because of its lower T_m_. This also reduces the issue of non-specific amplification as usually seen in a conventional asymmetric PCR. In short, the initial exponential phase aims at exploiting the region of interest on target DNA and accumulating the maximum number of ds-amplicons, whereas the linear amplification phase aims at producing ssDNA form the ds amplicons. As a consequence of these events, very high amounts of ss-amplicons can be generated from a very low concentration of template, which can be used for the microarray applications.

The specificity and amplification efficiency of the designed primers is assessed by real time PCR using SYBR green dye and 10-fold dilution of *Staphylococcus aureus* genomic copies (Supplementary Figure S2). A standard curve with the Ct values of these amplicons showed an amplification efficiency of 99.6% with an *R* ^2^ value of 0.994 (Supplementary Table S2 and Supplementary Figure S3). The melt curve analysis with the generated amplicons showed homogenous amplification an sharply defined melting peaks at 86°C (Supplementary Figure S4).

### Guard banding studies for amplification cycles

A study on the required number of amplification cycles at each stage (exponential and linear) of the amplification is performed to find a balance between these two steps. Goal is that the balanced system should be able to efficiently amplify a target concentration of 10 genomic copies of *Staphylococcus aureus.*

To assist the study, a theoretical model with standard PCR amplification kinetics is formulated to estimate the number of amplicons generated for a primer concentration at ideal conditions. According to this model in Table 2, the concentration of the un-extended primer is adjusted such that it is available only during the exponential phase to amplify as little as one genomic copy of the template. According to the AELA-PCR principle the concentration of un-extended primer should not be an important factor as they are not active in the linear phase of the amplification. But it is used here as a factor to estimate the required number of amplification cycles in the exponential phase to amplify the minimum possible amount of template DNA. Considering the first scenario (Case I in Table 1) when 0.1 uM of the un-extended primer is used, it is expected that the solution has 6.02 × 10^10^ molecules of un-extended primer per microliter and 1.20 × 10^12^ molecules in a 20 µl PCR mix. In an ideal case of exponential amplification without any errors, the respective un-extended primer molecules should be used up by 40.1 cycles (1 × 2^40.1^) of amplification with one genomic copy of template. Similarly, the same amount of un-extended primer molecules should be used up by 36.8 cycles (10 × 2^36.8^) and 33.5 (100 × 2^33.5^) cycles respectively for amplifying 10 and 100 genomic copies.

**TABLE 2:** A model to calculate the required amount of un-extended primer molecules and amplification cycles to amplify varied concentrations of initial template in an asymmetric PCR.

Case	Un-extended primer [µM]	Un-extended primer molecules/µl	Un-extended primer molecules	Required exponential amplification cycles to completely use un-extended primer		
			20 µl—PCR mix	1 genomic copy	10 genomic copies	100 genomic copies
I	0.1	6.02 × 10^10^	1.20 × 10^12^	40.1	36.8	33.5
II	0.2	1.20 × 10^11^	2.41 × 10^12^	41.1	37.8	34.5
III	0.5	3.01 × 10^11^	6.02 × 10^12^	42.4	39.1	35.8

In agreement with this model, starting the amplification with 0.1 µM of the un-extended primer is estimated that the system would require about 36 exponential cycles to amplify 10 genomic copes of template. Based on these numbers, the exponential amplification cycles are guard banded at 30, 35, and 40 cycles by using 0.3 µM of un-extended primer. The linear phase amplification cycles are studied from 15 to 30 cycles with an interval of five cycles by keeping the exponential cycles as constant. To assist this, a very high amount of extended primer concentration is used.

**FIGURE 3:**
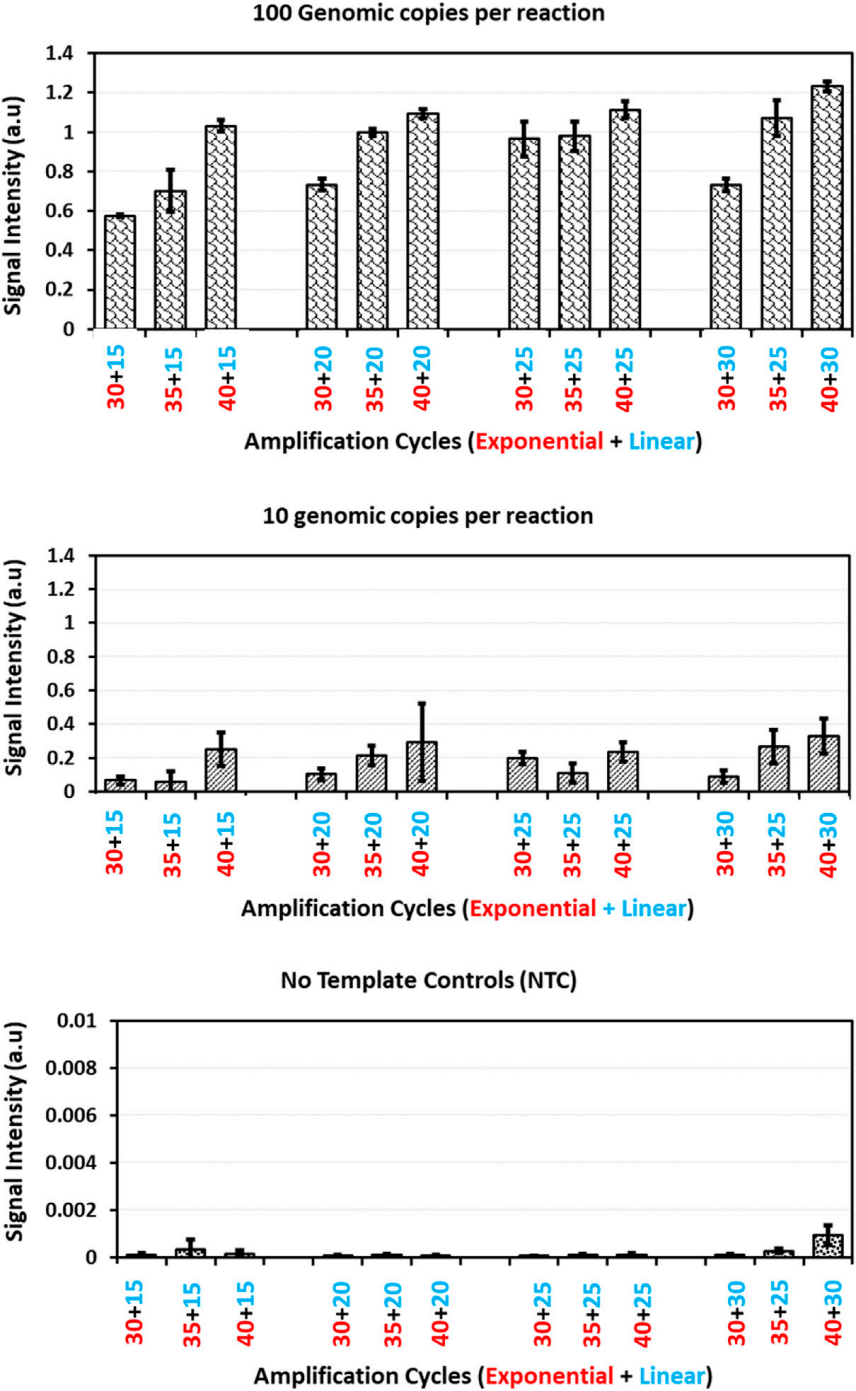
Guard banding of amplification cycles: Signal intensities obtained for *Staphylococcus aureus* probe after amplification of *Staphylococcus aureus* copy number dilutions (100, 10 genomic copies and no template control) with different amplification cycles patterns and hybridization onto microarray. The error bars represent standard deviation across five replicates for each dilution in a respective amplification method.

This change in exponential amplification cycles at a constant linear amplification cycles showed quite significant improvement in the biochip hybridization signal intensities with both 100 and 10 genomic copies per amplification reaction (Figure 3). On the other side, comparing the datasets with varying linear amplification cycles for a respective exponential cycle number showed small improvement in the hybridization signal intensities. This can be understood and explained by the functionality of exponential and linear procedures. With a change in exponential amplification cycles, the change in the number of amplicons generated in ideal conditions would be a factor of 2^n^ whereas for linear amplification the change would be a factor of 2xn. In this case, a change in five amplification cycles, the amount of amplicons generated in exponential amplification would translate to an increase of 32 times of initial concentration whereas this would be only 10 times with linear amplification. This explains the significance observed with a change in exponential amplification and with a change in linear amplification. This suggests the importance of initial exponential amplification cycles required for the linear phase to be able to generate detectable amounts of single stranded amplicons.

**FIGURE 4:**
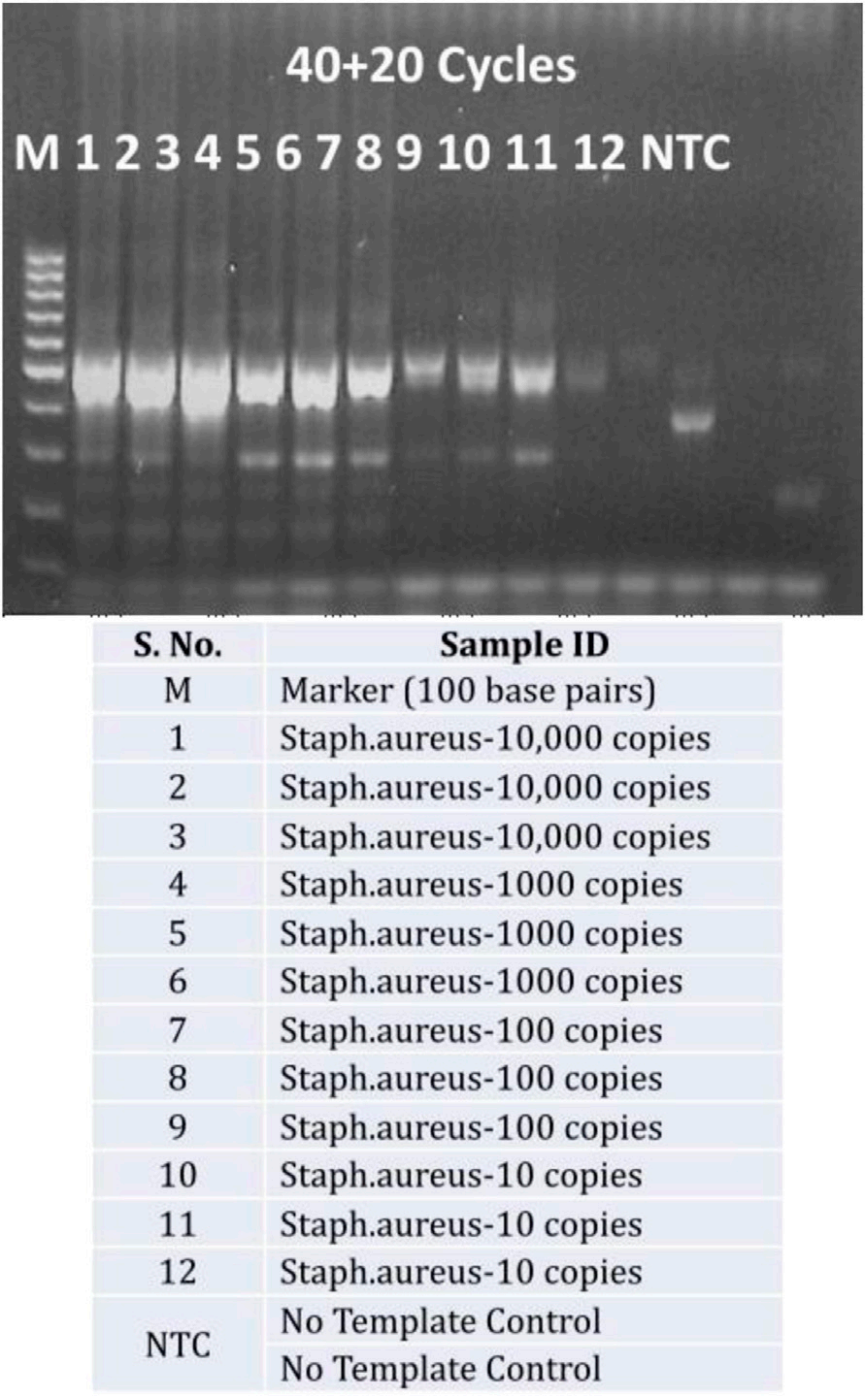
Unspecific amplification with excessive cycling: Agarose gel electrophoresis of PCR products from 40 exponential and 20 linear amplification cycles of *Staphylococcus aureus* copy number dilutions (10,000 copies to 10 copies per reaction and No Template control-NTC) against a 100 bps marker.

A closer look at increasing the exponential amplification cycles to 40 shows higher standard deviation, indicating the variability of observed hybridization signal intensities. This can be translated as variable amounts of available PCR products for hybridization. This has been explained previously by many authors that excessive cycling can mis-prime the amplification and can generate unspecific amplicons (Roux, 1995; Ruiz-Villalba et al., 2017; Sze and Schloss, 2019; Walker et al., 2020). The gel electrophoresis images of these runs (Figure 4) also showed extra bands with lower molecular weight than the expected (470 base pairs), specifically at low template concentrations and No Template Controls (more data available in Supplementary File as Agarose gel electrophoresis images of amplification cycles under Supplementary Table S1 and Supplementary Figure S1).

This is dependent of the primer concentration and the availability of target to non-target template in the reaction but this does not mean that higher primer concentration would avoid the issue. Conversely increased primer concentration could provoke the formation of primer dimers in the reaction, despite having stringent primer design strategies (Kainz, 2000; Jansson and Hedman, 2019). One of the major reasons for unspecific amplification with excessive cycling is due to the annealing of PCR products 3′-OH to the available genomic template (Bell et al., 1991) or to itself. These 3′-OH ends are then extended by polymerases and terminated randomly resulting in products with random lengths. These factors encouraged the decision to use 35 cycles of exponential amplification.

Similarly, experiments with a change in linear amplification cycles beyond 25 cycles showed increased standard deviations and also did not show significant improvement in the observed signal intensities. Moreover, unnecessary cycling is a waste of raw materials and time. Considering these factors, the combinations of 35 exponential and 20 linear amplification cycles is chosen as standard and further studies were done to compare the AELA-PCR method with established PCR techniques.

### Comparison of PCR methods

The performance of AELA PCR is evaluated through juxtaposition with other PCR methods used in microarray applications. A comparison experiment with symmetric PCR and standard asymmetric PCR is performed. For a symmetric PCR, equal concentrations of primers with almost similar melting temperatures (T_m_) were used. The same primers were used for asymmetric PCR but the primers were added at a ratio of 20:1 (Forward primer: reverse primer). For both of these setups, the amplification was performed for 50 cycles. For AELA PCR, primers were designed as discussed above and amplification is performed according to the thermal profile with 35 exponential and 20 linear amplification cycles. A genomic copy number dilution series of *Staphylococcus aureus* DSMZ pure culture DNA, spiked with human genomic DNA is used as target DNA for amplification. The presence of human genomic DNA as background in the pure culture target DNA mimics the standard infectious blood extracted bacterial DNA sample in clinical settings. The generated amplicons were hybridized to the fabricated 3D microarray in each well of a 96-well microtiter plate. After incubation, the array is washed and scanned to look for bound amplicons and processed for analysis.

**FIGURE 5:**
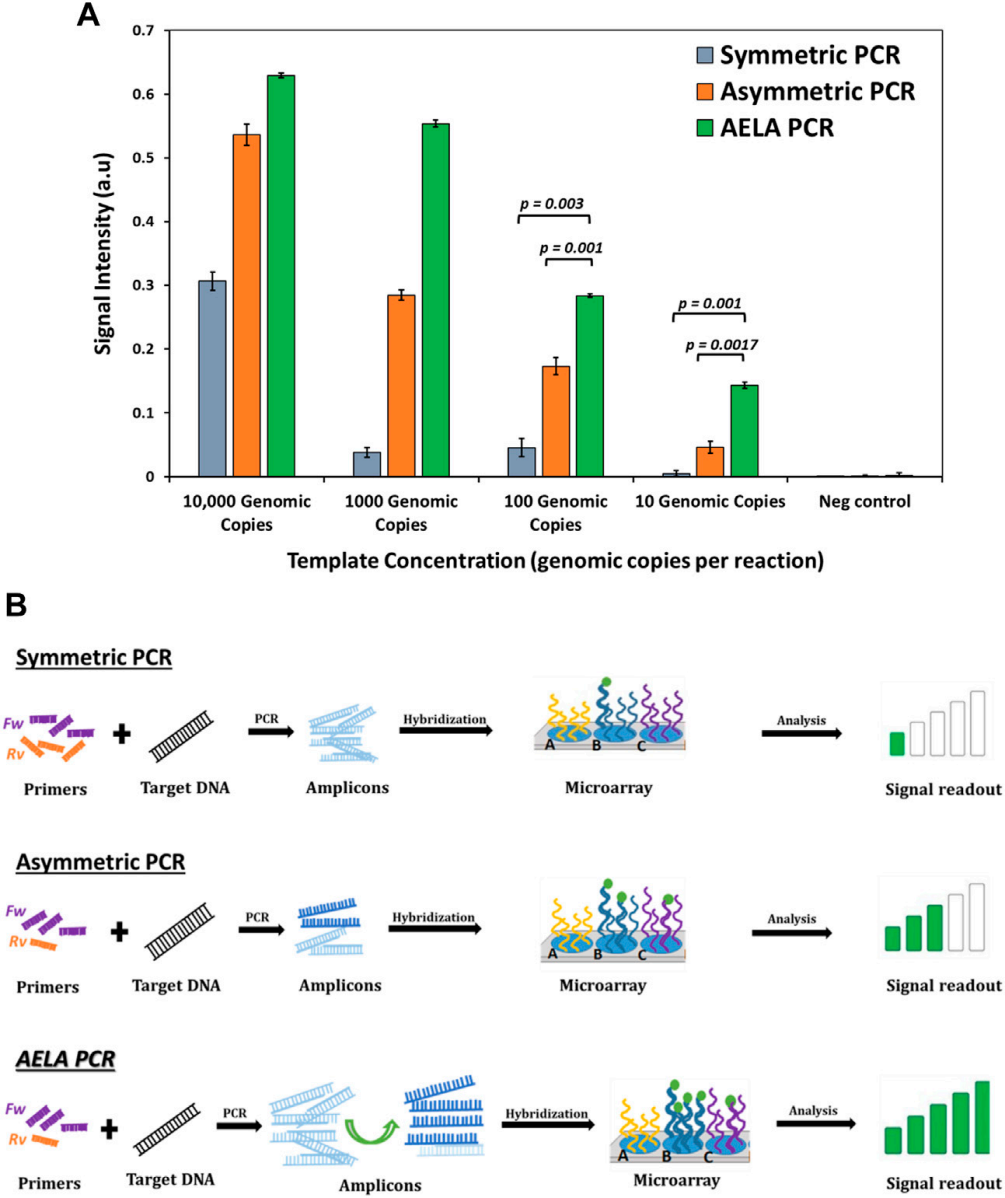
Comparison of conventional, asymmetric and AELA PCR: **(A)** Signal intensities for *Staphylococcus aureus* probe obtained after amplification of *Staphylococcus aureus* copy number dilutions with different PCR profiles and hybridizing the amplicons on to the microarray. The “*p*-values” shows the significance for *t*-test for null hypothesis, which means any value ≤ 0.05 is significant. **(B)** Schematic representation of microarray signal readout with different PCR profiles. The body of primer sequence used for the three PCR profiles is the same but as shown in Figure 1 the AELA PCR has an extended self-complimentary sequence to its 5′ end. The primers are used at equal concentrations for the symmetric PCR but used at 20:1 concentrations for asymmetric and AELA PCR reactions.

As seen in Figure 5A, the observed signal intensities from hybridization of amplicons generated from symmetric PCR show a signal significantly above background only at high template concentration of 10,000 genomic copies in the amplification reaction. The signal intensities for lower template concentrations such as 1,000 and 100 genomic copies per reaction show signal strengths only slightly above the no template control. Amplifying further lower concentration, 10 genomic copies per reaction with symmetric PCR amplification did not show any detectable signal intensities. The signal intensities from hybridization of amplicons from conventional asymmetric PCR showed great improvement compared to that of symmetric PCR. Even at a low template concentration of 10 genomic copies per reaction, a small detectable signal is observed significant signal above background. However, compared to the conventional asymmetric PCR, the AELA PCR showed several times higher signal strengths especially at lower template concentrations.

As a symmetric PCR contains equimolar concentration of primers and the annealing temperature is within the T_m_ range of the primers, both are active in the amplification and generate amplicons at an exponential rate resulting in a predominate formation of double stranded amplicons. When they are hybridized on the microarray, they have to diffuse to the spots of the 3D microarray, melt and reanneal to the immobilized capture probe (Figure 5B). Because of the length of the complementary strands, they generally have higher reannealing efficiency to each other compared to the reannealing to capture probe which generally is a shorter oligonucleotide. Due to this predominate self-reannealing of amplicons from symmetric PCR, very few of the total number of amplicons generated can hybridize to the capture probes and a rather large number of amplicons is required to give a signal. From Figure 5A, the number of surface-hybridized amplicons generated at high concentration of template DNA will be larger compared to the reaction with low concentration of template DNA. This explains the presence of signal intensities with 10,000 genomic copies and absence of detectable signal with 10 genomic copies per amplification reaction.

In an asymmetric PCR, the amplification occurs exponentially until the limiting primer is completely consumed. Then it switches to linear amplification with the available excess primers which can generate amplicons from one strand of target DNA. This results in the generation of a mixture of double and single stranded amplicons. As shown in Figure 5B, when these are hybridized on to the microarray, the presence of single stranded amplicons increases the chances of binding to complementary capture molecules on the microarray. This in-turn generates higher signal intensities with asymmetric PCR compared to symmetric PCR.

The AELA PCR system is designed to facilitate the generation of single stranded amplicons. As discussed before, the initial 35 cycles of amplification results in generation of a large number of amplicons which is followed by 20 cycles of linear amplification so that in the end most of the DNA generated in the process is single stranded (Figure 4B). This results in efficient binding of amplicons to the complementary capture probes on microarray, which reflects high signal intensities even at low template availability.

### Validation of AELA-PCR

**FIGURE 6:**
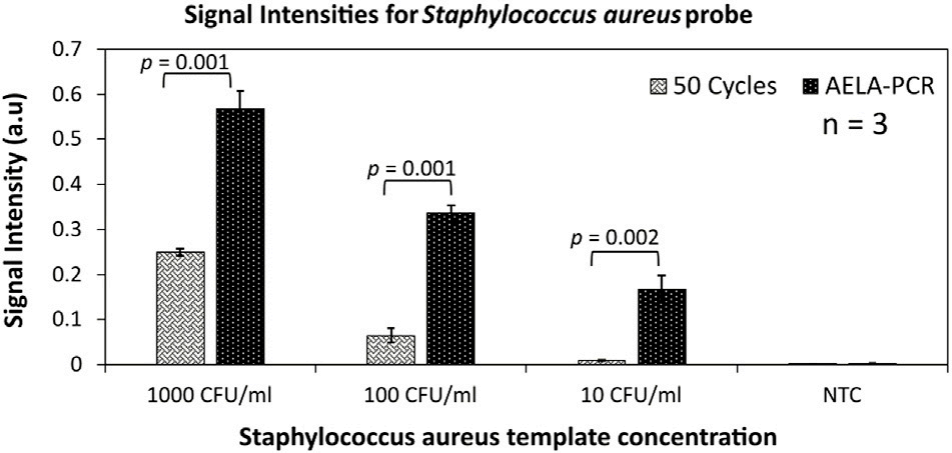
Validation of AELA PCR: Signal intensities for *Staphylococcus aureus* probe obtained after amplification with 50 asymmetric PCR amplification cycles and AELA-PCR of extracted *Staphylococcus aureus* template from spiked blood culture samples and hybridizing the amplicons. The error bars represent standard deviation across three replicates (*n* = 3) for each dataset and CFU/ml represents Colony Forming Units per ml of blood. The “*p*-values” shows the significance for *t*-test for null hypothesis, which means any value ≤ 0.05 is significant.

Even after an efficient extraction of bacterial DNA from infected or spiked blood, it has to be understood that the extract contains many blood-based components along with the target bacterial DNA. These blood components at most cases are major concerns for PCR inhibitions and strongly limit the control of conventional asymmetric PCR. When a spiked blood sample with *Staphylococcus aureus*, which is chosen here as a demonstration case, is amplified performing conventional asymmetric PCR and AELA PCR, in which higher signal intensities were obtained in guard banding studies. Despite of the background blood components, the AELA PCR showed almost 250 times higher signal intensities compared to a conventional 50 cycles asymmetric PCR (Figure 6). This shows that the AELA PCR is more specific and sensitive in detection of target DNA with a microarray.

### Limit of detection

**FIGURE 7:**
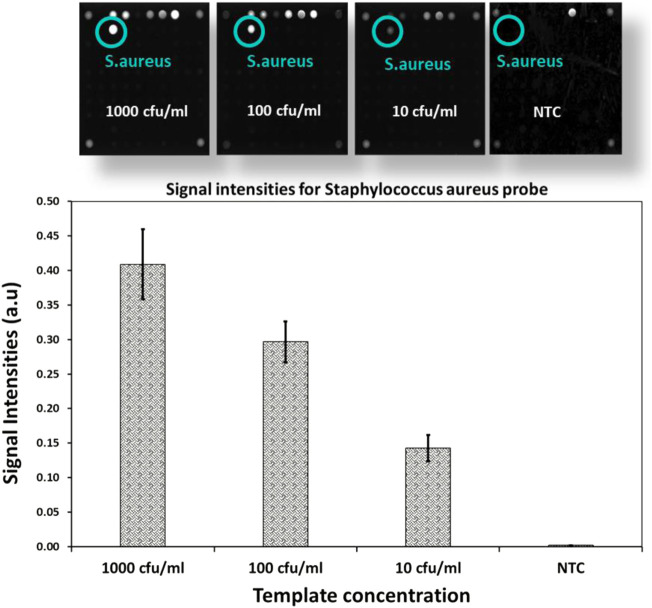
LOD of AELA PCR and Microarray system: Microarray images and bar chart with signal intensities obtained after the hybridization of the dilution series of spiked *Staphylococcus aureus* sample (NTC represents No Template Control). Microarray image shows signal intensities for different controls used in the assay (not discussed in this manuscript) and *Staphylococcus aureus* probe. The *Staphylococcus aureus* probe is circled in green and annotated at each concentration.

To determine the limits of the assay, *Staphylococcus aureus* DNA extracted from spiked blood samples with concentrations of 1,000 cfu/ml down to 10 cfu/ml is used. A sample without spiked bacterial template is used as no template control (NTC). These dilution series are amplified with AELA-PCR for 10 repetitions, performed by three different persons in two different laboratories over 5 days and hybridized onto arrays on five separately printed plates (Figure 7 and Table 3). All of the 10 repetitions showed a visible signal above the background (at least 10 times higher) at the lowest concentration (10 cfu/ml) tested.

**TABLE 3:** Signal intensities and standard deviation of *Staphylococcus aureus* probe at different concentrations (NTC represents No Template Control).

	*Staphylococcus aureus* probe	
Concentration	Signal intensity	Standard deviation
1,000 cfu/ml	0.409	0.051
100 cfu/ml	0.297	0.030
10 cfu/ml	0.143	0.019
NTC (Blank)	0.002	0.000

The LOD, for an analyte was determined according to CLSI, document EP17-A and recommendations specified by Burd, 2010 according to.

$${LoB=mean}_{blank}+1.645\left({SD}_{blank}\right)$$ (1)

$$LoD=LoB+1.645\left({SD}_{low\;concentration\;sample}\right)$$ (2)

The observed signal intensity for 10 cfu/ml of spiked *Staphylococcus aureus* amplified with AELA-PCR is 0.143 and the calculated signal intensity at this respective concentration is 0.033 (Table 4). Which means, observed signal intensity values are about 4.3 times (more than 3SD) greater than required for concentration of 10 cfu/ml. This demonstrates that the Limit of Detection (LOD) for *Staphylococcus aureus* is 10 CFU/ml or lower which is superior to data reported in the literature (Banada et al., 2012; Perker et al., 2018; Zhang et al., 2021).

**TABLE 4:** Limit of Blank (LOB) and Limit of Detection (LOD) calculated for all the corresponding probe for *Staphylococcus aureus*.

Spot ID	Limit of the blank (LOB: Signal intensity)	Limit of detection (LOD: Signal intensity) at 10 cfu/ml
*Staphylococcus aureus* probe	0.002	0.033

## Conclusion

AELA-PCR represents a simple and efficient PCR methodology to generate single stranded amplicons for microarray applications. In contrast to standard asymmetric PCR methods, AELA is much more efficient in generating ssDNA through a two-step approach where only the temperature profile is varied. In the first step, the initial exponential amplification of target DNA generates rapidly a large number of amplicons from the region of interest. The exponential amplification step is followed in the second step by a linear phase of amplification to generate rapidly a large number of ss-amplicons. Using a one-step labelling and amplification strategy with a labelled primer for amplification directly labelled amplicons are generated. This can greatly reduce the assay time for microarray applications. Compared to the reported methodologies in the literature and existing commercial techniques for generating single stranded amplicons, AELA PCR shows greater efficiency with good reproducibility in the generation of ssDNA. Irrespective of the primer concentration, the generation of single stranded amplicons can be realised based on differences in the primers melting. Using identical conditions in microarray applications, a factor of 200 fold higher sensitivity compared to conventional PCR could be demonstrated at the example of *Staphylococcus aureus* and sensitivity levels as low as 10 cfu/ml could be reached. The overall amplification procedure is simple, flexible and fit-for-all. This simplified amplification procedure makes the AELA-PCR system as promising candidate for amplification methodology in generating single stranded amplicons and for microarray applications.

## Data Availability

The original contributions presented in the study are included in the article/Supplementary Material, further inquiries can be directed to the corresponding author.
