# Supplementary material for: An advanced and efficient asymmetric PCR method for microarray applications
Source: Front Bioeng Biotechnol. 2022 Nov 30;10:1045154. doi: 10.3389/fbioe.2022.1045154 (PMC9748121; doi:10.3389/fbioe.2022.1045154)
Supplement: Supplementary file 1 [file DataSheet1.PDF]

Supplementary data/Appendix:

Agarose gel electrophoresis images of amplification cycles:

The PCR products from guard banding studies of amplification cycles is ran on 2% agarose gels assisted by bromophenol blue dye.

S. No.	Sample ID
M	Marker (100 base pairs)
1	Staph.aureus-10,000 copies
2	Staph.aureus-10,000 copies
3	Staph.aureus-10,000 copies
4	Staph.aureus-1000 copies
5	Staph.aureus-1000 copies
6	Staph.aureus-1000 copies
7	Staph.aureus-100 copies
8	Staph.aureus-100 copies
9	Staph.aureus-100 copies
10	Staph.aureus-10 copies
11	Staph.aureus-10 copies
12	Staph.aureus-10 copies
NTC	No Template Control
	No Template Control

Supplementary table 01: sample layout for Agarose gels

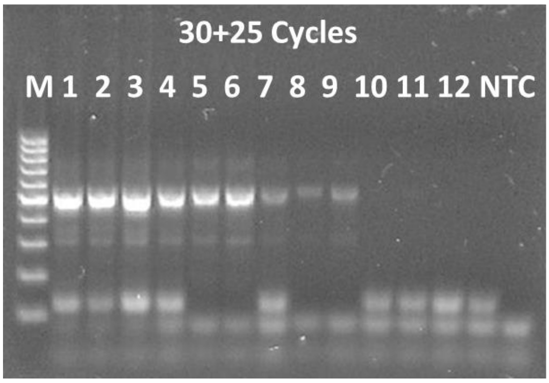

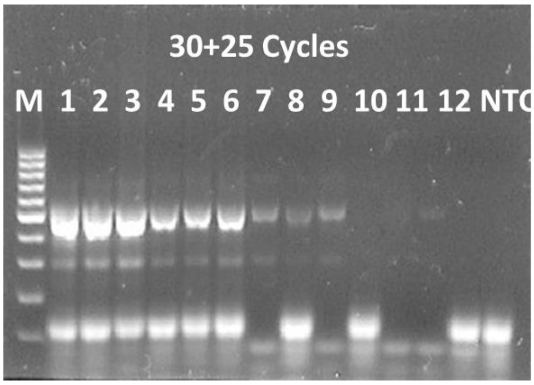

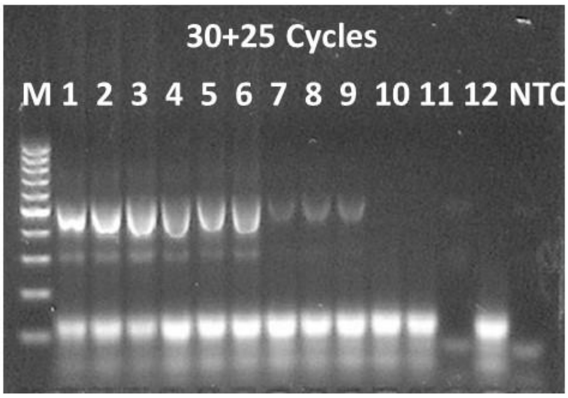

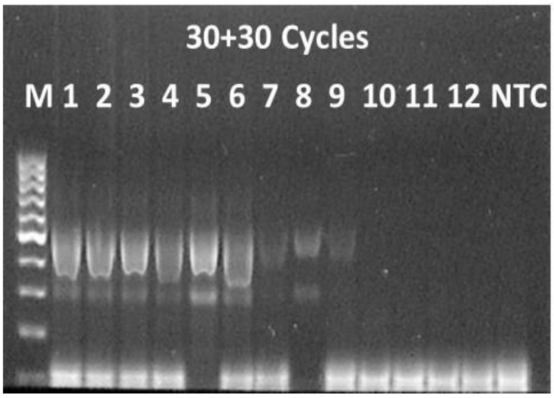

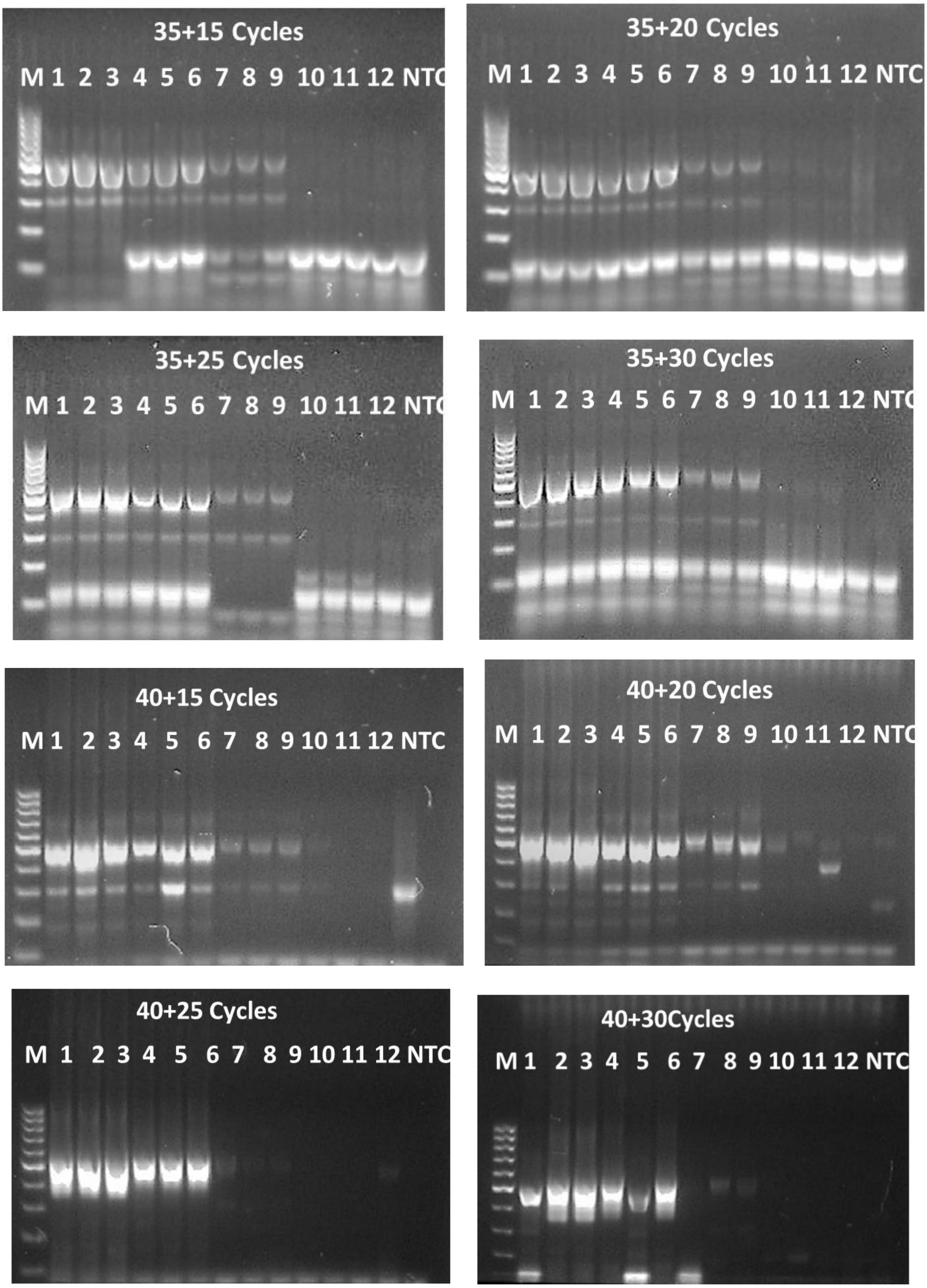

Supplementary figure 01: 2% Agarose gel electrophoresis images for guard banding amplification cycles. All the gels have same sample layout as mentioned in supplementary table 01 and ran against a 100 base pairs marker (M). The expected amplicon size from the primer pair used for the study is about 500basepairs.

Efficiency of Primers:

The capability of the primers to bind specifically to the target sequence and amplify properly with no or less unspecific products determines the sensitivity levels of an amplification-based system. This has been assessed with real-time amplification methodology aided by SYBR green dye. The primers designed for AELA-PCR are used to amplify 10-fold genomic copy number dilutions of *Staphylococcus aureus*.

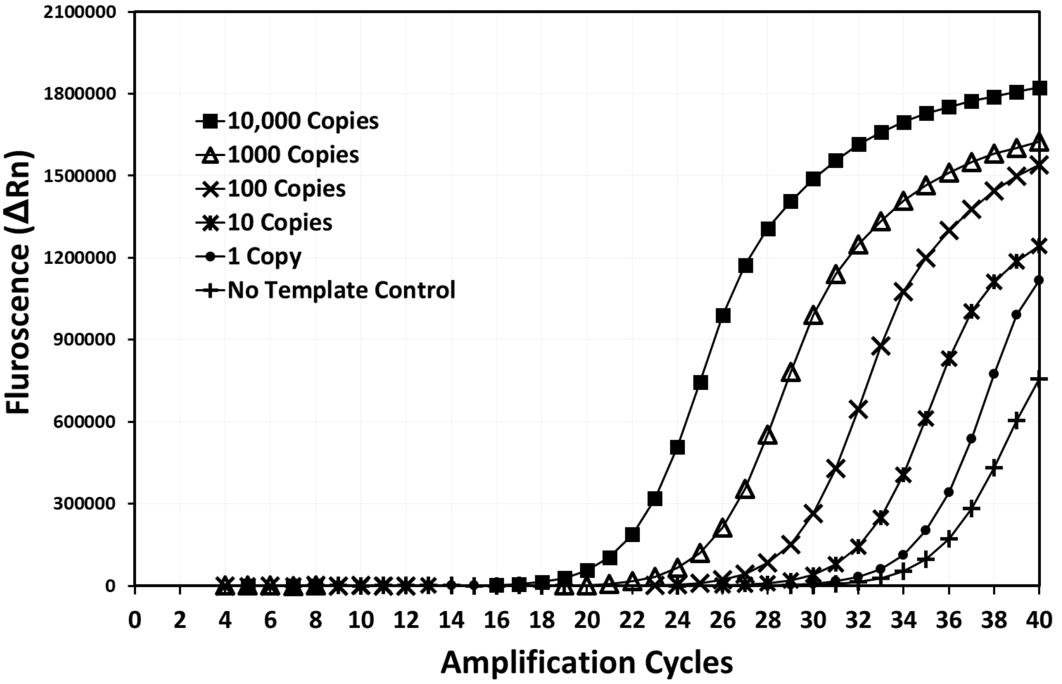

Supplementary figure 02: Analysis of the efficiency of primers using real-time PCR with SYBR green dye. The amplification curve obtained with fluorescence generated by amplification with primers using *Staphylococcus aureus* genomic copy number dilutions. The threshold line is manually set at 200000 (a.u) for generating the cycle threshold (C_T) values.

As seen in supplementary figure 02, the data from real time amplification plot obtained by amplification of *Staphylococcus aureus* genomic copy number dilutions with the primers designed for this work showed good reproducibility across the replicates and consistent Ct difference between each dilution step. The threshold line is manually set at 200000 (a.u) for better Ct (threshold cycle) values.

Supplementary table 02: The mean cycle threshold (C_T) values with standard deviations (SD) obtained from the real-time PCR assay for primers with amplification of *Staphylococcus aureus* template.

S.No	Template concentration per reaction	C_T Mean	C_T SD
1	10,000 Copies	20,9	0,10
2	1000 Copies	24,0	0,09
3	100 Copies	28,1	0,05
4	10 Copies	31,4	0,33
5	1 Copies	33,9	0,47
6	No Template Control	35,7	2,06

With these Ct values, a standard curve is generated with respect to the log concentration of the template.

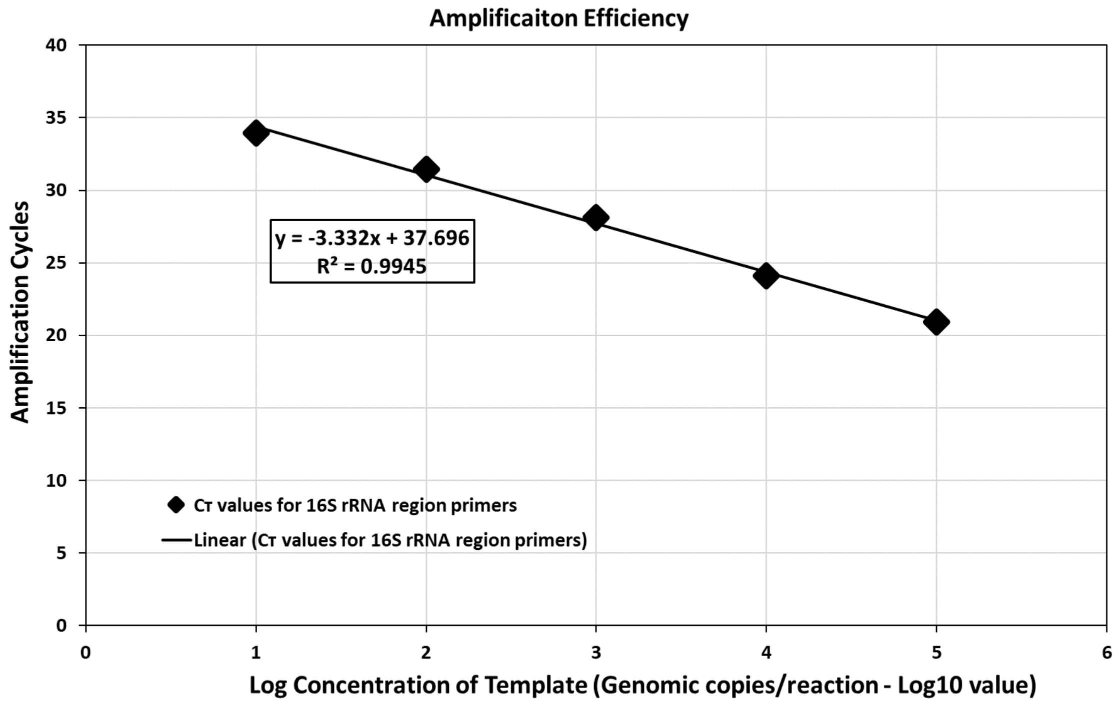

Supplementary figure 03: A standard curve generated against the log concentration of template and amplification cycles with the Cycle threshold (C_T) values obtained from real-time PCR with the primers.

The slope obtained from the standard curve is then translated to the efficiency (E) of the amplification (M. Kubista et al 2006).

Efficiency (E) = $10^{(-1/slope)}$ (S. 1)

E for 16S rRNA primers = $10^{(-1/-3.332)}$

Efficiency of 16S rRNA primers (E) = 1.99

Percentage Efficiency (%E) = (E-1) x100 (S. 2)

%E for 16S rRNA region primers = (1.9958-1) x100

Percentage Efficiency of 16S rRNA region primers (%E) = 99.6

The percentage efficiency indicates the deviation in quantity of the PCR product from the theoretical doubling with each cycle. A slope of –3.3 ±10% reflects an efficiency of 100% ±10%. This means that efficiency between 90 to 110% is considered as acceptable. An efficiency of 99.6% for 16S rRNA primers would indicate the ability of them to bind the target amplicon and amplify specifically at good rates.

The other important factor to further understand the specificity of primer amplification is to check the possibilities of primer dimers which can significantly reduce the efficiency of the amplification. This is assessed instantaneously by performing melt curves (supplementary figure 04) of the amplicons generated. Primer dimers are said to be the products of unspecific amplification generated during the annealing and elongation events of the amplification. These may start generating as soon as the amplification reagents are mixed together at room temperature even if the primers are designed with necessary care. Any such events would significantly increase the chances for unspecific amplification. When generated, these unspecific amplicons can also compete with the desired PCR products, limits the dynamic range and efficiency of amplification.

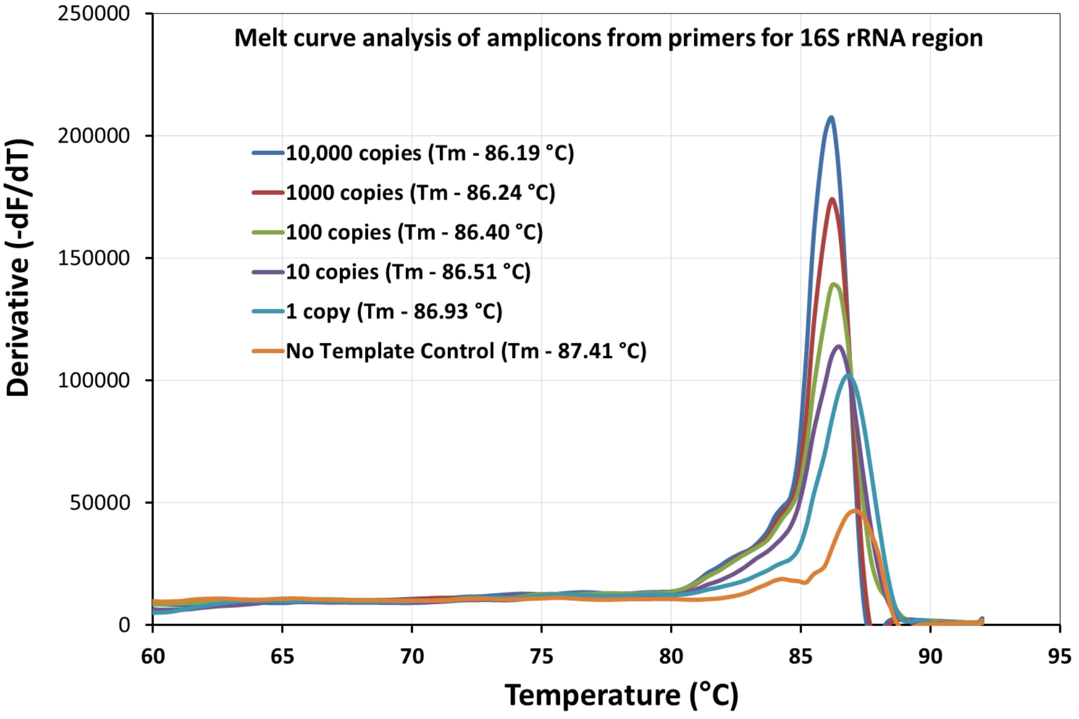

Supplementary figure 04: Melting curves generated with the amplicons from real-time PCR with primers.

The melting curve analysis performed after the amplification with a set of primers, have the ability to identify both the desired PCR amplicon and the unspecific amplification resulted from primer dimers. Pure and amplicons generated from homogenous amplification generally produce unique and sharply defined melting peak behaviour. The amplicons generated from unspecific primer dimer artefacts are generally smaller and melt at lower temperature than the desired PCR product, resulting in broader secondary melting peaks. The melting curves generated from amplifying *Staphylococcus aureus* with primers as represented in supplementary figure 04 show sharply defined single peaks indicating that there is one type of amplicon generated, which should be the desired amplicon. These peaks are consistent as one bunch which also reflects that a single type of amplicon is been generated every time.

Calculation of genomic copy numbers:

The copy number dilutions are prepared by calculating molecular weight from the genome size of the respective strain. The following supplementary table 03 provides an example for Staphylococcus aureus DSM 20231 strain used in the study.

Supplementary table 03: Calculations for generating copy number dilutions.

Species	Genome size (bp) (from NCBI)	Mol wt (g/mol) [Genome size X 660]	Mol wt in pg/ 1 copy	Mol wt in pg/ 10000 copies	Stock (pg/µl)	Vol. req. to make 10000 copies from stock	Water to add (final 50ul)	Reference species
Staphylococcus aureus	2782560	1836489600	0.0031	30.5065	200	7.63	42.37	Staphylococcus aureus subsp. aureus DSM 20231

The table below outlines primers used, their ratio and thermal profiles used in the experiments to show the superiority of AELA PCR.

Supplementary table 04: Primers information and cycling parameters used in PCR's comparison study

<table>
<tr><th>PCR type</th><th>Primer sequence</th><th>Primers Ratio</th><th colspan="7">Thermal profile</th></tr>
<tr><td rowspan="2">Symmetric PCR</td><td>Fw - TGAACGCTGGCGGCGTGCCTAATAC</td><td rowspan="2">1:1</td><td>Denaturation</td><td colspan="3">Annealing 1</td><td colspan="2">Annealing 2</td><td>Extension</td></tr>
<tr><td>Rv - CACGGTCGTCGGCGCCATTATG</td><td>95°C 2min</td><td>95°C 20sec</td><td>58°C 15sec</td><td>72°C 40sec</td><td colspan="2">-</td><td>72°C 2min</td></tr>
<tr><td rowspan="2">Asymmetric PCR</td><td>Fw - TGAACGCTGGCGGCGTGCCTAATAC</td><td rowspan="2">20:1</td><td rowspan="2">95°C 2min</td><td rowspan="2">95°C 20sec</td><td rowspan="2">58°C 15sec</td><td rowspan="2">72°C 40sec</td><td rowspan="2" colspan="2">-</td><td rowspan="2">72°C 2min</td></tr>
<tr><td>Rv - CACGGTCGTCGGCGCCATTATG</td></tr>
<tr><td rowspan="2">AELA PCR</td><td>Fw - CAGCGTTCA TGAACGCTGGCGGCGTGCCTAATAC</td><td rowspan="2">20:1</td><td rowspan="2">95°C 2min</td><td rowspan="2">95°C 20sec</td><td rowspan="2">58°C 15sec</td><td rowspan="2">72°C 40sec</td><td rowspan="2">95°C 20sec</td><td rowspan="2">72°C 50sec</td><td rowspan="2">72°C 2min</td></tr>
<tr><td>Rv - CACGGTCGTCGGCGCCATTATG</td></tr>
</table>
